# Editorial: Proteomics of Microbial Human Pathogens

**DOI:** 10.3389/fmicb.2016.01742

**Published:** 2016-11-04

**Authors:** Nelson C. Soares, German Bou, Jonathan M. Blackburn

**Affiliations:** ^1^Division of Chemical and Systems Biology, Department of Integrative Biomedical Sciences, Faculty of Health Sciences, Institute of Infectious Disease and Molecular Medicine, University of Cape TownCape Town, South Africa; ^2^Servicio de Microbiologia-Instituto de Investigación Biomédica, Complejo Hospitalario Universitario A CoruñaA Coruña, Spain

**Keywords:** proteomics, mass spectrometry, microbes, bacteria, virulence, pathogens, protein posttranslational modifications

Despite remarkable advances in treatment and prevention, infectious diseases remain amongst the leading causes of death worldwide, particularly in the developing world, and drug resistant pathogens are ominously on the rise. By way of one specific example, the global incidence of human tuberculosis (TB) disease—caused by infection with the pathogen *Mycobacterium tuberculosis*—is estimated to be ~9 million new cases (~0.1% of the global population) per annum, causing 1.5–2 million deaths per annum and with an estimated 450,000 people developing multi-drug resistant tuberculosis each year); moreover, the WHO estimates that ~1/3rd of the World's population carries a latent *M. tuberculosis* infection, thus representing a huge reservoir of potential future TB cases. In the context of this Research Topic, it is also relevant that the burden of TB disease is very uneven globally, with certain countries in Africa having a much higher incidence than observed in the developed world—for example, in South Africa the national incidence of TB disease is around 1% of the population and a single city, Cape Town, reports more cases of TB annually than the whole of North America and Europe combined. Furthermore, roughly 21% of all deaths in South Africa are associated with TB disease today, despite proactive efforts at TB control since the beginning of the twentieth century and despite the TB control program in Cape Town achieving 75% case finding and 85% completion rates for smear positive disease. Similarly depressing statistics abound for numerous other infectious diseases in the developing world, with disease burden being driven by both microbial adaption (to drug resistance and to differing ecologies), as well as by the emergence of new, often zoonotic pathogens (e.g., ebola and zika viruses). According to the World Health Organization (WHO), infectious diseases today account for more than 70% of premature deaths across 22 African countries (Who, 2014), with co-infections, for example with HIV or helminths, being rife. It is therefore critical that we develop new molecular knowledge now that will inform new strategies in the global fight against human microbial pathogens—including those that are not prevalent in the developed world—with the use of state-of-the art “omics” technologies set to take center-stage through providing a detailed understanding of the mechanistic basis of pathogenicity and by creating a comprehensive description of the molecular interplays that mediate host-pathogen interactions.

During the last two decades, powerful high throughput research platforms—namely genomics, transcriptomics, and proteomics—have contributed significantly to our understanding of the life styles of pathogenic microbes, including important aspects of host interactions and infection (Raskin et al., 2006; Merhej et al., 2013; Yang et al., 2015). Of all the omics platforms, proteomics is perhaps the research platform that has undergone the greatest transformation, including a structural transformation, as it evolved from initial two-dimensional gel electrophoresis (2-DE gel) based workflows to sophisticated shotgun proteomics analysis, capable of generating comprehensive data with a coverage of individual proteomes that begins to approach that of corresponding genomics/transcriptomics datasets now. Modern mass spectrometry (MS)-based approaches today enable the detection and relative/absolute quantification of several thousand proteins in a single run, providing the opportunity to make a major impact on our general understanding of microbial pathogens. The goal of this Research Topic is thus to provide a survey of recent mass spectrometry-based research on the proteomics of human pathogens, which term we use broadly to refer to microorganisms that cause disease in humans, including *inter alia* virus, bacteria, fungi, and protozoa.

In this Research topic, we have particularly sought to highlight the powerful synergies that can be found between the fields of proteomics and microbiology, as exemplified by the review of Baarda and Sikora, which outlines the treasure hunt for counter-measures against old disease (Baarda and Sikora). Their review starts by acknowledging early contributions of 2-DE gel approaches, and how this approach implicated novel proteins—such as peroxidoxin, outer membrane protein Rmp, and the 50S ribosomal proteins L7/L12—in the *Neisseria gonorrhoeae* acquired resistance to spectinomycin. They then highlight how the employment of recent quantitative proteomics (SILAC, iTRAQ, and iCAT) has illuminated the pathways utilized by *N. gonorrhoeae* to adapt to different lifestyles, including during host interaction itself. A similar trend, suggesting that progression in the field of proteomics strongly augments classical microbiological research on microbial pathogens, is also evident in several others reviews (Ravikumar et al.; Pérez -Llarena and Bou; Soufi and Soufi), as well as opinion articles, in this Research Topic (Soares and Blackburn).

Elsewhere in this Research Topic, Perez-Llarena and Bou describe proteomics as a tool to study bacterial virulence (Pérez-Llarena and Bou) and, following this lead, this Research Topic carries several original reports that employ proteomics based approaches to investigate different aspects of bacterial/fungi virulence, including biofilm formation (Arnal et al.), motility (Merino et al.), virulence factors (Diehl et al.), altered virulence between strains (Peters et al.), and determinants that mediate host interaction in *Candida albicans* (Marin et al.). For example, Peters et al. used a combination of discovery and targeted liquid chromatography-tandem mass spectrometry (LC-MS/MS)-based proteomics to compare the proteomes of six clinically relevant mycobacterial strains within the *M. tuberculosis* complex (MTBC) (Peters et al.); MTBC members show a high degree of genetic conservation (~99.9%), yet clinically they exhibit differing pathogenicity and virulence. Peters et al. identified an average of 3290 protein groups for each MTBC organism, corresponding to >80% coverage of the theoretical proteomes; thereafter, the authors identified quantitative differences between strains for specific proteins that could be linked to enhanced bacterial fitness in the more virulent W. Beijing lineage of *M. tuberculosis* (Peters et al.).

This Research Topic also includes a number of manuscripts that explore novel applications of mass spectrometry within microbial research (Hartmann et al.); (Potgieter et al.); (Soares and Blackburn) (Soufi and Soufi), providing evidence that proteomics has now evolved into a robust platform capable of generating comprehensive datasets comparable in size and utility to those described in genomic studies and enabling the application of mass spectrometry-based proteomics to genome reannotation and refinement (Krug et al., 2011). For example, in this topic, Potgieter et al. report the results of a proteogenomic analysis of *M. smegmatis*, integrating mass spectrometry-based proteomics with genomic six frame translation and *ab initio* gene prediction databases to identify a total of 2887 open reading frames (ORFs), including 2810 ORFs previously annotated to a reference protein and 63 ORFs not previously annotated to any reference protein (Potgieter et al.).

Finally, several papers in this Research Topic have described the enormous contribution of proteomics to the knowledge-based regarding bacterial protein post translation modifications (PTMs) and their intimate association with virulence (Ravikumar et al.); (Calder et al.); (Pérez -Llarena and Bou); (Soares and Blackburn); (Soufi and Soufi) and it will be interesting to see whether future investigation of bacterial PTMs provide a means to discover targets for novel therapies (Soufi and Soufi). In an Opinion article, we challenge researchers in the field to join efforts and to take advantage of recent advances in mass spectrometry instrumentation and in the development of targeted quantitative workflows that enable detection and accurate quantification of bacterial PTMs (Soares and Blackburn) since we believe that this represents a golden opportunity to access the dynamics of bacterial PTMs at sites of infection and that, through this, researchers will gain meaningful insights into the functional role of such PTMs during host-pathogen interactions.

## Conclusion

Overall, this Research Topic demonstrates the huge potential for modern mass spectrometry-based (phospho)proteomics to yield major breakthroughs in our understanding of host-pathogen interactions. Therefore, it seems reasonable to suppose that as proteomics-based experimentation is married ever more closely with biologically relevant models of human microbial disease, the analytical power of quantitative mass spectrometry will yield testable hypotheses about key molecular host-pathogen interactions and might identify new candidate drug or vaccine targets. However, in order to reach this Holy Grail, the burgeoning microbial proteomics field will need to not only infer but then experimentally validate true biological significance from amongst the vast datasets generated. Tighter integration and iteration between computational modeling of proteomic data and model-driven further experimentation, including use of cell biology, imaging and CRISPR technologies therefore seems likely to represent the future.

## Author contributions

NS and JB wrote the original draft of the paper, read and edited the final draft. GB critically discussed the content of the manuscript.

### Conflict of interest statement

The authors declare that the research was conducted in the absence of any commercial or financial relationships that could be construed as a potential conflict of interest.
